# Congenital laryngo-tracheo-esophageal clefts: updates from a quaternary care pediatric airway unit

**DOI:** 10.1007/s00405-023-08263-8

**Published:** 2023-10-11

**Authors:** Alessandro Ishii, Emeline Christophel, Madeleine Chollet, Kishore Sandu

**Affiliations:** 1 Department of Otorhinolaryngology, Lausanne University Hospital, Lausanne, Switzerland; 2 Department of Anaesthesia, Lausanne University Hospital, Lausanne, Switzerland

**Keywords:** LTEC, Laryngo-tracheo-esophageal cleft, Endoscopic surgery, Open surgery

## Abstract

**Purpose:**

To review the operative techniques, outcomes, and complications following surgery in pediatric patients with laryngo-tracheo-esophageal clefts (LTEC). We describe a new *combined* approach to treat long LTECs.

**Methods:**

Twenty-five patients underwent surgical repair for LTEC from March 2012 to July 2022 at our hospital. Every patient underwent a diagnostic endoscopy under general anesthesia and spontaneous ventilation to assess the LTEC and synchronous aero-digestive comorbidities/malformations. All patients underwent at least one surveillance endoscopy after the repair at our institution.

**Results:**

The patients had multiple other malformations, specifically gastro-intestinal, synchronous airway, and cardiac. The cleft distribution according to the modified Benjamin and Inglis classification was type I (*n* = 5, 20%), type II (*n* = 6, 24%), type IIIa (*n* = 8, 32%), type IIIb (*n* = 4, 16%), and type IVa (*n* = 2, 8%). The median follow-up was 44.6 months. Five patients (20%) had undergone previous cleft corrective surgery(s). Seven patients (28%) had partial to complete breakdown of the repair, needing additional intervention(s), and two required a combined—open plus endoscopic repair. Preoperatively, most patients (*n* = 18, 72%) needed a feeding assistance. At latest follow-up, feeding assistance was weaned off in 13 out of 18 patients, which was a 72% improvement. Ten patients (40%) needed ventilation assistance before the surgery. Post-operatively, ventilatory assistance was weaned off in 6 patients, meaning a 60% improvement.

**Conclusion:**

LTEC are rare malformations, and their management needs precise diagnosis, appropriate surgical planning, and execution, and dedicated post-operative care. Primary and revision repair of long clefts with tracheal extension may require a combined approach.

## Introduction

Laryngo-tracheo-esophageal cleft (LTEC) is a rare malformation involving the aero-digestive tract. It was described for the first time by Richter in 1792 [1]. The exact incidence is still yet to be determined, although various authors range it from 1/10,000 to 1/20,000 patients [2]. The suggestion that a LTEC resulted from an interruption of the cephalad development of the tracheoesophageal septum was first introduced by Blumberg et al. [3]; however, the exact pathophysiological process is yet to be fully understood. Among the different classifications proposed, probably the most widely used is the one proposed by Benjamin and Inglis, which stratifies 4 different types of LTEC [4]. A modified classification that allows distinction between clefts passing through the partial or entire length of the posterior cricoid plate and longer clefts extending further into the extra- and intra-thoracic trachea and bronchi was then developed [5].

Complete division of the posterior cricoid plate by the cleft and its further tracheobronchial extension makes the laryngo-tracheal framework unstable and the airway severely compromised. Endoscopic cleft repair has now been accepted as the standard treatment for low-grade LTEC (types I–II) [6]. However, difference of opinion continues to remain for the higher grades of LTEC (III–IV). Open surgery has been the longstanding standard for treating high grades LTEC, but recently endoscopic treatment for selected cases has been proposed. Sandu and Monnier succeeded in treating endoscopically four cases of type III LTEC [5], and these results were reproduced by Leishman et al. [7]. The most important complication following an endoscopic repair of a long LTEC is the tissue dehiscence occurring either at the lower end of the cleft, thus leading to development of a tracheoesophageal fistula or a proximal defect leading to cleft recurrence.

This report supplements to our experience in the management of LTEC, in addition to the previous publications by our unit [5, 7]. We discuss a combined surgical approach to repair long clefts with tracheal extension and propose technical points to improve surgery-specific results.

## Methods

### Study design and setting

All patients operated after March 2012 were prospectively entered into a LTEC registry since our units’ last publication on this subject [7]. For this study, we reviewed cases entered in this registry up to July 2022. A total of 25 patients underwent surgical repair for LTEC during this time frame at our hospital. After obtaining institutional board review approval and local ethics committee authorization (CER-VD 2020-00641), records of all patients were analyzed.

### Patients

We included all pediatric patients with the diagnosis of LTEC, except those who were already described in our previous articles [5, 7]. All patients were referred to our unit from other Swiss and European hospitals and had at least one post-LTEC repair endoscopy at our institution. Feeding evaluation (functional oral intake scale FOIS) [8], Modified Barium Swallow (MBS) and diagnostic endoscopy were already done at these centers and the patients were referred to us purely for surgical repair of the cleft. Information regarding the subsequent follow-up was obtained by electronic mail exchanges with the referring doctors and parents/guardians of the patients. A follow-up registry is maintained in our unit and is regularly updated depending on the patient information received from these centers. The information provided in this report covers the entire study period and up to the most recent follow-up of the patient by his treating doctor.

### Diagnostic endoscopy

**Fig. 1:**
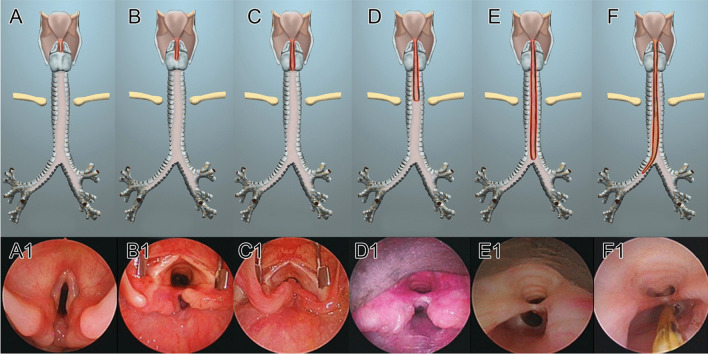
Modified Benjamin Inglis classification of laryngo-tracheo-esophageal Clefts (LTEC). **A–A1** Type I: Supraglottic interarytenoid cleft extending up to the vocal cords. **B–B1** Type II: partial cricoid cleft extending beyond the vocal cords. **C–C1** Type IIIa: total cricoid cleft. **D–D1** Type III b: LTEC extending into the extrathoracic trachea. **E–E1** Type IVa: LTEC extending to the carina. **F–F1** Type IVb: LTEC extending into one main stem bronchus (MSB) (right MSB in the photograph). Adapted from Sandu and Monnier [5]

Every patient underwent a diagnostic endoscopy under general anesthesia and spontaneous ventilation to assess the LTEC as well as other aero-digestive comorbidities/malformations. A laryngeal spreader was used to see the lower extent and the length of the cleft. The *modified* Benjamin–Inglis system was used for staging the cleft [5]. This classification system (Fig. 1) staged the clefts depending on its extension through the cricoid (partial or complete); the trachea (extra- and intra-thoracic) and the bronchi. The referring institutions followed similar endoscopy technique and cleft classification.

### Anesthesia

Tubeless and total intravenous anesthesia plays an important role during the endoscopic closure of the LTEC. The larynx and the trachea were sprayed with local anesthesia 0.5% Novesine (Oxybuprocaine) and it was repeated every 30–45 min. Dexmedetomidine (DMT) was started at the concentration of 1 µg/0.1 ml with a bolus of 4 µg/kg/hr over 10 min, following which, continuous perfusion of DMT (2–4 µg/kg/hr) and remifentanil (0.1 µg/kg/min) was administered. A Portex Blue Line endotracheal tube (ETT) maintaining oxygenation of 6L/min was passed nasally and placed in the hypopharynx, away from the laser field. During the entire procedure, the patient had cardiac monitoring, CO_2_–O_2_ saturation and the BIS (BiSpectral Index, Medtronic) recording. A second small-for-age Portex blue line soft ETT was kept ready for an oro-tracheal intubation without removing the suspension laryngoscope. The tube was passed into the trachea under endo- or microscopic guidance using a microlaryngeal curved crocodile forceps until the child regained adequate saturation.

### Surgical technique

**Fig. 2:**
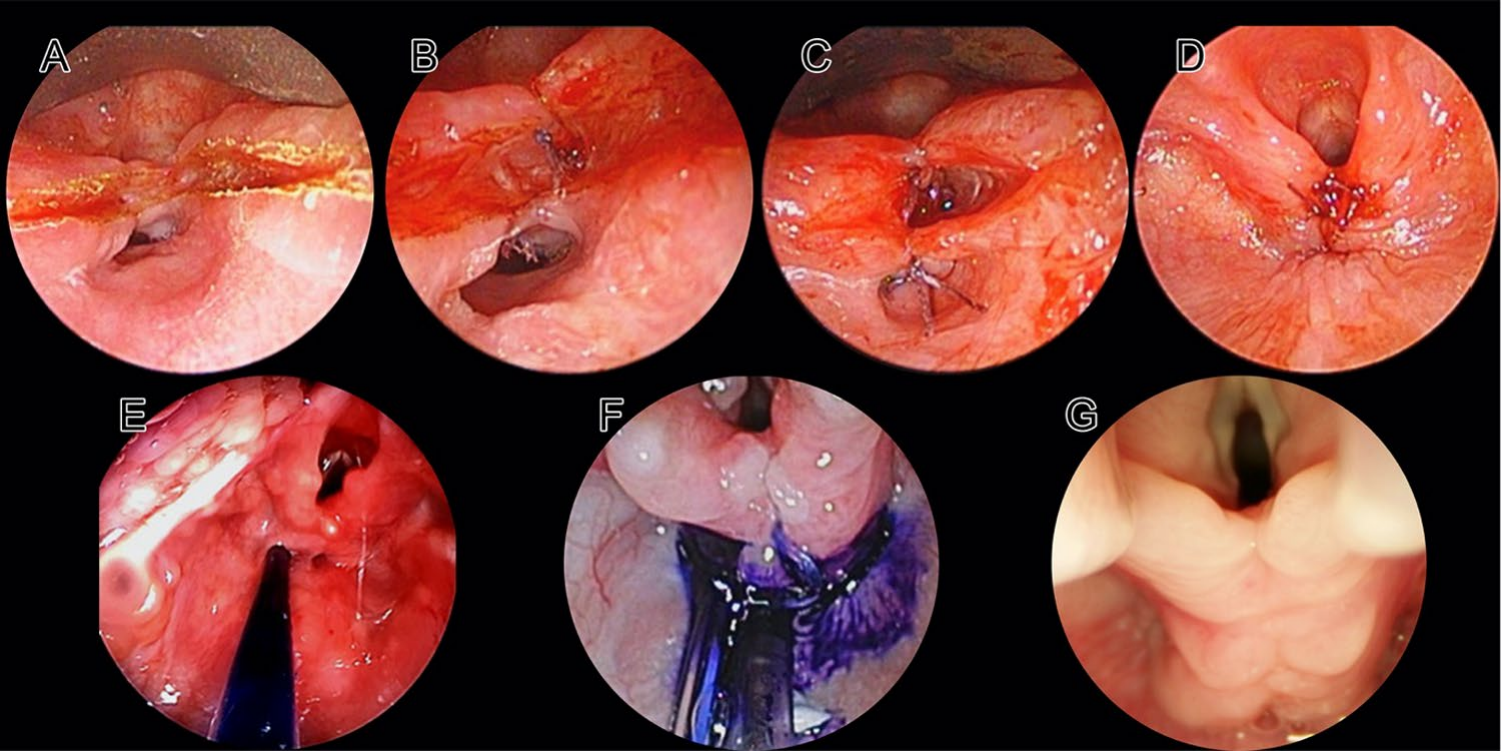
Endoscopic repair of type III b LTEC. **A** CO2 (carbon dioxide) laser incision of the cleft and creation of the laryngo-tracheal (LT) and pharyngo-esophageal (PE) mucosae. **B** The cleft closure is done in layers starting from apex of the cleft in the cranio-caudal direction. **C** Two-layer (LT and PE) closure of the cleft. **D** Closure of the cleft is done up to just below the cuneiform cartilages. **E** Methylene Blue (MB) test. Naso-gastric tube is passed into the esophagus to instill the MB. **F** Over-spilling of MB and its passing into the airway must be avoided. Simultaneously, the airway and the cleft closure integrity is checked. **G** Post-operative result after 2 years

Endoscopic approach (Fig. 2): An adequate-size Parsons laryngoscope with side slot (ref. 8576 C, D, E) was selected and inserted through the vocal cords to spread them apart and expose the most distal limit of the cleft and simultaneously visualizing the esophagus, and the trachea.

We used the Lumenis Duo CO2 laser (ultrapulse mode, 1 W, sharp focus 250 microspot) mounted on a Leica PROVido microscope for the endoscopic intervention. We started at the distal extremity of the cleft and, coming from caudal to cranial ends of the cleft, created two mucosal layers (trachea–laryngeal (TL) and eso-pharyngeal (OP)) up to just below the cuneiform cartilages on either side of the cleft. Hemostasis was achieved using a fine monopolar cautery point and adrenaline-soaked cotton pledgets. We used 5.0 Vicryl (TF-1 Plus, Ethicon, ref. V133H needle size 10 mm in most cases) or 7.0 Polydioxanone (Ethicon, PDS-II, needle size 9.3 mm in very small infants and neonates, ref. Z1701E) to suture the two mucosal layers, beginning from the caudal end and progressing up to the cranial end of the cleft. The sutures in both the mucosal layers were tied toward the esophageal side, and the sutures on the trachea–laryngeal layer were cut close to the knot. The laryngoscope was progressively withdrawn to place more cranial sutures. Fibrin glue (Tisseel) was placed between the two mucosal layers just before tying the most cranial suture. Depending on the cleft length, total of 2 (type I)–20 (types III and IV) sutures were used to close the cleft.

**Fig. 3:**
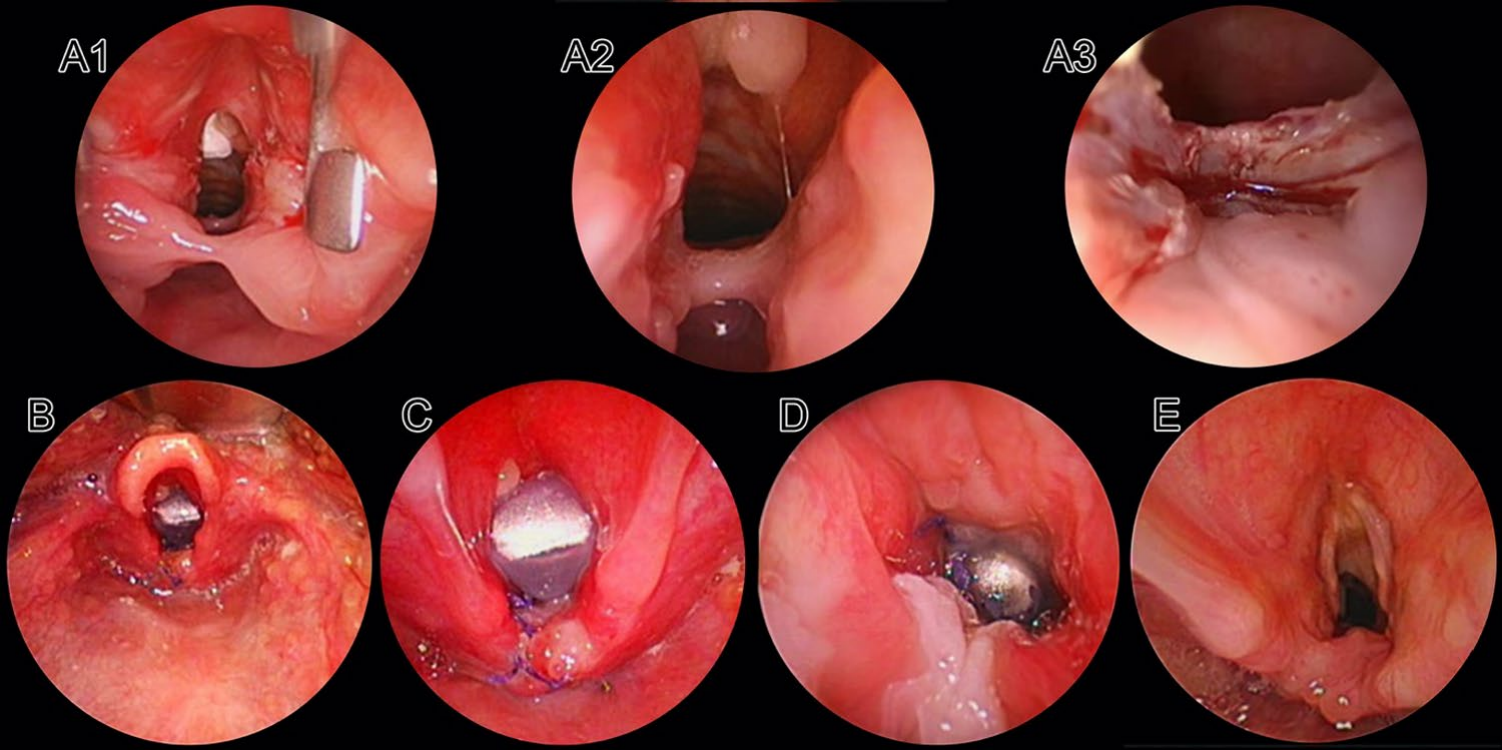
Combined approach for laryngo-tracheo-esophageal Cleft (LTEC) closure. **A1–3** Incomplete breakdown following an endoscopic closure of a type III b LTEC. **A1** interarytenoid mucosal band. **A2** Incomplete breakdown of the cleft. **A3** CO2 (carbon dioxide) laser is used to divide the interarytenoid band and then proceed with *tattooing* of the cleft. **B-C** Proximal end of a *small-for-age* LT mold stent. **D** Distal end of the LT mold seen through the tracheostomy site. **E** Post-operative photo after 12 months

Combined approach (Fig. 3): This approach was used for long clefts (types III and IV) either as an upfront primary surgery or after a repair breakdown. If the patient had a prior LTEC surgery in another center, surgery at our institution was performed after a waiting period of at least 4–6 months following the prior surgery. All our patients had a prior tracheostomy. The surgery started with a microscopy-assisted ‘laser tattooing’ of the cleft, and then followed by an open repair. Thin mucosal attachments in a near-complete breakdown of a previously operated long cleft were laser divided. Laser marking of the cleft has the advantage of clearly demarcating the two mucosal layers (TL and OP), which could then be identified during the open repair. The next step was to perform a full laryngofissure. The OP mucosal closure was made from caudal to cranial ends of the cleft using 5.0 Vicryl and the sutures are cut toward the esophageal side. A rib cartilage graft was harvested and sculpted to have cranial and caudal perichondrial extensions. The cartilage was fixed to replace the dehiscent posterior cricoid plate, and the perichondrial extensions remained interposed between the OP and TL mucosal layers. The TL layer was closed with separate 5.0 Vicryl sutures knotted toward the airway. A small-for-age Monnier’s’ laryngo-tracheal LT-Mold avoiding decubitus on the cleft sutures was placed and doubly fixed (in the supraglottis and trachea) using 3.0 Prolene. The laryngofissure was closed using multiple intermittent 4/5.0 Vicryl sutures.

### Post-operative management

Following an endoscopic repair, the patients were given non-invasive ventilation, transferred to the pediatric intensive care unit, and received antibiotics and proton pump inhibitors. When the ventilatory assistance was waived and no signs of complications were present, the patient was transferred to a standard room. First LTEC repairs and re-operated patients were similarly managed in the post-operative setting.

All patients underwent a surveillance endoscopy under general anesthesia and spontaneous respiration at one-week post-surgery and a dilute methylene blue (MB) test was performed. An adequate-size suction catheter was passed through the nose and then under endoscopic vision into the esophagus. Methylene blue was injected into the catheter that was withdrawn up to the cricopharyngeal opening and its spill over into the airway was avoided. A mild laryngeal massage was given to the neck to allow distribution of the instilled MB over the entirety of the cleft. The integrity of cleft closure was checked by passing a long telescope in the airway up to the lower limit of the cleft. Residual cleft at the proximal end and any fistulization of MB was noted.

In the combined approach, the LT-Mold was kept for 6–8 weeks and removed under suspension laryngoscopy. Integrity-check of the cleft closure was done as described earlier.

Some patients had MBS prior to their referral to our center. Swallow therapists of our hospital evaluated patients before and after the surgery and oral feeds were started only after confirming optimal cleft closure during post-operative endoscopy. No MBS was performed in the post-repair period, swallowing evaluation was done by FOIS analyses.

## Results

From March 2012 to July 2022, a total of 25 patients were treated for LTEC at our clinic, with a median follow-up of 44.6 months (11 m–9 years). Most of them were males (*n* = 19, 76%). The cleft distribution according to the modified Benjamin and Inglis classification was, type I (*n* = 5, 20%), type II (*n* = 6, 24%), type IIIa (*n* = 8, 32%), type IIIb (*n* = 4, 16%), type IVa (*n* = 2, 8%). The mean age at first surgery at our clinic was 2 years and 20 days old (range from 30 days to 10.2 years). Patients overview is mentioned in Table 1.

**Table 1:** Sociodemographic, comorbidities, malformations, and surgical history of the patient population

No	Cleft type	Age at surgery	Gender	Comorbidities	Other malformations	Airway surgery history	Gastrointestinal surgery history	Cardiovascular surgery history	Prior LTEC repair attempt
1	I	0 years 1 months 25 days	Male	Preterm (36 weeks 0 days)	Omphalocele, dysmorphic facies, placental mosaicism, ASD	No	Omphalocele correction	No	No
2	I	0 years 0 months 29 days	male	Preterm (37 weeks 5 days), intrauterine growth retardation, bi-chorial bi-amniotic twin pregnancy	Complete bilateral cleft lip-palate, penis-scrotal hypospadias, polymalformative syndrome (fusion of thoracic vertebral bodies,dysmorphic facies)	No	No	No	No
3	I	1 year 11 months 24 days	Male	G6PD deficiency	PDA, hernia humbilicalis	No	No	PDA correction	No
4	I	6 years 9 months 19 days	Female	Tracheobronchomalacia	Diaphragmatic hernia, supra-subglottic-stenosis grade II-III	LTR-APCCG	No	No	No
5	I	0 years 10 months 26 days	Male	None	Tracheal bronchus (right)	No	No	No	No
6	II	0 years 7 months 20 days	Male	None	Esophageal atresia, ASD, TEF	TEF correction	Esophageal atresia correction, multiple esophageal dilatation (6x)	No	No
7	II	1 year 5 months 23 days	Male	Trisomy 21, Hirschprung's disease, tracheobronchomalacia	Cricoid hypoplasia	No	ILEOSTOMY and continuity restauration	No	No
8	II	0 years 4 months 16 days	Male	None	None	No	No	No	No
9	II	0 years 2 months 10 days	Male	None	None	No	No	No	No
10	II	3 years 4 months 28 days	Male	None	None	No	No	No	No
11	II	1 year 11 months 19 days	Male	None	None	No	No	No	No
12	IIIa	0 years 2 months 7 days	Male	Preterm (36 weeks 2 days), peritonitis on jejunum perforation	None	No	Laparotomy for jejunum perforation	No	No
13	IIIa	3 years 2 months 20 days	Male	None	None	No	NO	No	Yes (endoscopic)
14	IIIa	1 years 11 months 2 days	Female	Tracheobronchomalacia	None	No	No	Aortopexy	Yes (endoscopic)
15	IIIa	0 years 1 months 9 days	Male	None	None	No	No	No	No
16	IIIa	10 years 2 months 15 days	Female	Preterm (26 weeks 2 days), IVF tri-chorial tri-amniotic triplet, hyaline membrane disease, relapsing pneumonia, pulmonary hypertension	VACTERL, anal atresia, recto-vaginal fistula, hernia hiatalis, pharyngeal web	No	Ano-rectal malformation correction, colostomy, continuity restauration, Nissen fundoplication, gastrostomy	no	Yes (6 × endoscopic)
17	IIIa	2 years 7 months 4 days	Male	OSAS, tracheobronchomalacia	Esophageal atresia	No	Esophageal atresia correction	No	Yes (endoscopic)
18	IIIa	2 years 6 months 14 days	Female	Preterm (28 weeks 2 days), twin pregnancy, hyaline membrane disease, growth retardation, chronic respiratory failure on pulmonary hypertension, mental retardation	Esophageal atresia, TEF, pig bronchus, microgastria, subglottic stenosis grade I	TEF correction	Esophageal atresia correction, gastrostomy Nissen fundoplication, pyloroplasty	no	Yes (2 × endoscopic)
19	IIIa	2 years 0 months 30 days	Male	Left vocal fold palsy	Subglottic-stenosis grade I (Cotton-Myer), uvula bifida	No	No	No	No
20	IIIb (2 rings)	2 years 7 months 24 days	Male	Trisomy 21	Esophageal atresia	No	Esophageal atresia correction	No	No
21	IIIb (3 rings)	7 years 0 months 14 days	Male	None	Left lung agenesis	No	Nissen fundoplication	No	No
22	IIIb (6 rings)	0 years 3 months 22 days	Female	Tracheobronchomalacia	VSD, ASD	No	Nissen fundoplication, Kader gastrostomy	No	No
23	IIIb (1 ring)	0 years 4 months 22 days	Male	None	None	No	No	No	No
24	IVa	0 years 0 months 30 days	Male	Preterm (36 weeks 0 days), chromosome 13 duplication, polyhydramnios	None	No	No	No	No
25	IVa	0 years 1 months 8 days	Female	Preterm (34 weeks 0 days), triplet pregnancy	PDA	No	No	No	No

*ASD* atrial septal defect, *G6PD* glucose-6-phosphate-dehydrogenase, *LTR-APCCG* laryngo-tracheal reconstruction with anterior and posterior costal cartilage graft, *MSB* main stem bronchus, *OSAS* obstructive sleep apnea syndrome, *PDA* patent ductus arteriosus, *TEF* tracheoesophageal fistula, *VACTERL* vertebral defects, anal atresia, cardiac defects, trachea-esophageal fistula, renal anomalies and limb abnormalities, *VSD* ventricular septal defect

### Comorbidities and associated malformations

Seven patients were born preterm, with four of them from a multiple pregnancy (two each from a twin and a triplet pregnancy). The most common synchronous airway lesion was tracheobronchomalacia (5 patients; 20%). Four patients had a confirmed genetic syndrome (2 had trisomy 21, 1 had chromosome 13 duplication, and 1 had G6PD deficiency). Two patients had dysmorphic facies and one had VACTERL association.

There were multiple other malformations, specifically gastro-intestinal (*n* = 9, 36%, esophageal atresia, microgastria, diaphragmatic hernia, omphalocele, and umbilical hernia); airway malformations (*n* = 8, 32%, supraglottic stenosis, subglottic stenosis, tracheoesophageal fistula, cricoid hypoplasia, pig bronchus, left lung agenesis), cardiac (*n* = 4, 16%, atrial and ventricular septal defect, patent ductus arteriosus, patent foramen ovale), and genitourinary (anal atresia, recto-vaginal fistula, hypospadias). One patient had preoperative left vocal cord palsy.

The most frequent associated malformation was esophageal atresia (*n* = 4, 16%).

Seven patients had no malformation or comorbidity other than an isolated LTEC.

### Relevant past surgical history (Table 1)

Five patients (20%) had endoscopic repair attempted for LTEC prior to their referral to our clinic. 9 patients (36%) had past gastro-intestinal surgery, 3 (12%) had an airway surgery and 2 (8%) a cardiac surgery. 12 patients (48%) had no history of any prior surgery.

### Treatment and clinical outcome (Table 2)

**Table 2:** Surgery type details, complications, and functional outcomes

No	Type of initial surgery	Number of surveillance endoscopies	Complications	Number of correction surgery	Correction surgery	Pre-op feeding assistance (FOIS)	Post-op feeding assistance (FOIS)	Pre-op ventilation assistance	Post-op ventilation assistance	Follow-up (months)
1	Endoscopic	3	Residual cleft	1	Endoscopic	NGT (2)	Total oral (5)	BIPAP	Normal	48.4
2	Endoscopic	1	None	0	No	NGT (2)	Total oral (4.5)	Normal	Normal	29.1
3	Endoscopic	2	Residual cleft	1	Endoscopic	NGT (2)	Total oral (5)	Normal	Normal	23.9
4	Endoscopic	2	None	0	No	Total oral (4.5)	Total oral (5)	Tracheotomy	Tracheotomy	54.1
5	Endoscopic	1	None	0	No	total oral (4.5)	Total oral (5)	Normal	Normal	19.6
6	Endoscopic	7	None	0	No	PEG (3)	Total oral (5)	Normal	Normal	111.2
7	Endoscopic	2	Pneumonia	0	No	Total oral (4.5)	Total oral (6)	Normal	Normal	99.3
8	Endoscopic	1	None	0	No	NGT (2)	Total oral (4.5)	Normal	Normal	18.5
9	Endoscopic	2	None	0	No	NGT (3)	Total oral (4.5)	Normal	Normal	38.2
10	Endoscopic	4	Residual cleft	2	2 × endoscopic	Total oral (4)	Total oral (6)	Normal	Normal	41.1
11	Endoscopic	1	None	0	No	Total oral (4)	Total oral (6)	Normal	Normal	17.4
12	Endoscopic	1	None	0	No	NJT (2)	Total oral (5)	Normal	Normal	76.0
13	Endoscopic	1	None	0	No	PEG (3)	Total oral (5)	Tracheotomy	Normal	44.8
14	Endoscopic	1	None	0	No	PEG (3)	Total oral (4.5)	BIPAP	Normal	56.3
15	Endoscopic	1	None	0	No	NJT (2)	Total oral (5)	Normal	Normal	51.4
16	Endoscopic	1	Pulmonary hypertension post anesthesia	0	No	Total oral (4)	Total oral (5)	Tracheotomy	Tracheotomy	54.5
17	Endoscopic	2	Esophageal stenosis	0	No	Total oral (4.5)	Total oral (6)	Normal	Normal	51.1
18	Combined (endoscopic laser + cervicotomy + laryngofissure + PCCG)	3	Residual cleft	0	No	PEJ (3)	PEJ (3)	Tracheotomy	Tracheotomy	60.4
19	Combined (laser CO2 + LTR ACCG)	3	None	0	No	PEG (3)	PEG (3)	Tracheotomy	Normal	35.6
20	Endoscopic	1	None	0	No	NJT (3)	NJT (3)	Tracheotomy	Tracheotomy	28.1
21	Endoscopic	11	Minor-moderate residual cleft	3	2 × endoscopic, 1 × combined (laryngofissure laser CO2 + DS LTR (PCCG))	PEG (3)	Total oral (4)	Tracheotomy	Normal	44.3
22	Endoscopic	7	Residual cleft	3	2 × endoscopic, 1 × combined)	PEJ (2)	PEG (2)	Normal	Normal	13.5
23	Endoscopic	3	None	0	No	NGT (3)	total oral (4)	NIV	Normal	11.6
24	Open (cervicotomy + laryngofissure + PCCG)	1	Pneumonia, tracheal necrosis, death	1	Open	PEG (3)	death(-)	Normal	Death	(death)
25	Endoscopic	4	Pneumonia, bronchoaspiration, minor residual cleft	1	Endoscopic	PN (1)	total oral (4)	Normal	Normal	49.7

*ACCG* anterior costal cartilage graft, *BIPAP* bi-level positive airway pressure ventilation, *CO2* carbon dioxide, *DS LTR* double stage laryngo-tracheal reconstruction, *FOIS* Functional Oral Intake Scale, *NGT* nasogastric tube, *NIV* non-invasive ventilation, *NJT* nasojejunal tube, *PCCG* posterior costal cartilage graft, *PEG* percutaneous endoscopic gastrostomy, *PN* parenteral nutrition

Most of our patients were treated endoscopically (*n* = 22, 88%), and three patients had an upfront combined approach (of which 2 patients previously underwent unsuccessful endoscopic repair).

Most common complication observed was partial to complete cleft repair breakdown, seen in 28% of the patients (*n* = 7). Other complications observed were pneumonia (*n* = 3), pulmonary hypertension (*n* = 1), and cricopharyngeal narrowing (*n* = 1). 7 patients needed at least one additional corrective surgery for a failed cleft repair, of which 5 were corrected with one or more revision endoscopic procedures, and two patients received the combined approach. One patient (#24) had surgery-related death. No patient had post-operative vocal cord immobility, except the one who already had prior left vocal cord palsy.

Preoperatively, most patients (*n* = 18, 72%) needed feeding assistance in form of percutaneous endoscopic gastrostomy (*n* = 6), nasogastric tube (*n* = 6), jejunostomy (*n* = 5) and total parenteral nutrition (*n* = 1). After the LTEC correction, 13 out of 18 patients (72%) resumed adequate oral feeding. The mean FOIS scores pre- and post-surgery recorded were 3 and 4.5, respectively. In low-grade LTEC (I–II), the feeding assistance was weaned off in all 6 patients, as opposed to high-grade LTEC (III–IV), wherein the feeding assistance was weaned off only in 7 (63%) out of 11 patients (excluding patient #24).

Ten patients (40%) needed ventilation assistance before the surgery (7 had tracheostomy, 3 needed non-invasive ventilation). Post-operatively, ventilatory assistance was weaned out in 6 patients meaning a 60% improvement in comparison to the previous condition. Tracheostomy was closed in three out of seven patients, corresponding to a decannulation rate of 43%. The mean decannulation time post-surgery was 2 years, 6 months 28 days (*n* = 3, range 1 year 9 months 11 days–3 years 7 months 26 days). Three patients continue to have tracheostomy due to their complex comorbidities. One patient (#4) is undergoing active decannulation, but it is not yet achieved; hence, it is still considered with tracheotomy.

## Discussion

We report a new series of 25 patients who underwent treatment for LTEC at our center adding to our previous publications [5, 7]. In the current report, we show that the endoscopic cleft repair was optimal in most patients, improving their feeding and respiration compared to their preoperative status. It is important to note that early management of an anatomical cleft is critical to reduce aspiration risk, though swallowing issues may persist [9]. This should be discussed with the parents and caregivers to help guide their management expectations. Strychowsky et al. [9] demonstrated that patients with laryngeal clefts do have swallowing impairment in all phases of swallowing and this is not limited only to penetration and aspiration. The authors observed significant improvements in swallowing function in all surgically managed cleft patients, though without complete resolution of the swallowing issues. This is due to the high prevalence of comorbidities across all sub-groups of children with laryngeal clefts and the multifactorial etiology of their swallowing impairment. This was the case in our study population as well, especially in high-grade clefts (III–IV) where feeding assistance was still needed in 36% of the patients post-operatively.

Regarding post-operative complications, we had cleft repair failure in 28% of the patients, which slightly lower than 37% re-operation rate reported in the previous publication of our unit [7]. Other centers have similar reports: in an update from the Great Ormond Street Hospital in 2005, Kubba et al. [10] had 26% of their patients undergoing revision surgery because of a partial failure of the repair. In the 2010 series of Garabedian et al., 3 out of 11 patients necessitated a revision surgery, corresponding to a repair failure rate of 27% [11]. In a more recent study on high-grade LTEC (III–IV), Seidl et al. reported a recurrence of laryngeal cleft in 7 out of their 9 patients [12], which suggest that longer clefts are at higher risk of relapsing.

We recommend an endoscopic approach for LTEC types I–IIIa that have an adequate exposure of the entire length of the cleft, and in patients with optimal cardio-pulmonary reserves to tolerate general anesthesia with spontaneous breathing through the entire duration of the repair. The role of an experienced anesthesia team is critical. We present here a *combined surgical technique* to address long clefts extending deep into the trachea.

In our view to improve surgery-specific results, we would like to highlight few important points:

1. Type of the laryngoscope.
  We find the Parsons laryngoscope is best suited for the endoscopic repair of LTEC. Experts in this surgery have several sizes of this laryngoscope suiting specific patient requirement. We use this laryngoscope to spread the vocal cords apart, and to pass trans-glottic to expose the lowermost limit of the cleft, and to simultaneously expose the esophagus and the trachea before embarking on the cleft repair. The side slot allows a better manipulation of the needle carrier, while placing the sutures could be cumbersome in a closed laryngoscope. The side slot facilitates temporary tracheal intubation without removing the laryngoscope.
2. Laser versus cold steel.
  CO2 laser in an ultrapulse mode is an ideal cutting tool to *incise* the cleft, get into its deeper tissue and create two well-identifiable mucosal layers allowing a *layered closure*. Cold instruments would technically *excise* the cleft and sometimes overzealous removal of the mucosa will make the surgeon fall short of it, while making the repair sutures. Excising mucosal edges of a cleft using cold steel instruments, and mucosal denudation (laser- or cautery aided) require *mass closure* of the cleft as proposed by some authors [6, 13].
  The technique of using the laser and adequate knowledge of its tissue interactions must be well known to the surgeon to avoid complications. Several studies have shown that collateral tissue damage using CO2 laser is comparable to using cold steel instruments [14, 15].
  In the combined approach surgery, prior CO2 laser *tattooing of the cleft* and deepening the incision into the tissues help to correctly identify and repair the aero-digestive layers. When using the microscope, the surgeon visualizes the cleft in an axial plane and, therefore, can better prepare the distribution of the aero-digestive mucosa for the layered suturing. By this, we can avoid passing additional mucosa into the airway and causing a stenosis. In our experience, CO2 laser can be delivered by a microscope-mounted micromanipulator up to the proximal tracheal rings, and more distally using the fiber (Lumenis Duo).
3. Layered closure (LC) versus mass closure (MC) of the cleft.
  LC is described in this report and in previous publications of our unit [5, 7]. MC includes passing full thickness intermittent resorbable sutures through the mucosa denuded edge of the cleft and, therefore, duration-wise is rapid as compared to the layered closure. In the LC, first, the trachea–laryngeal mucosa is sutured together, and then bulkier passes are made to close the eso-pharyngeal mucosa. It is important to close the cleft up to just below the cuneiform cartilages to reduce chances of developing a residual cleft. While placing mass sutures, care should be taken not to be overzealous and include the arytenoid perichondrium in the sutures which could result in a tight posterior glottis and respiratory symptoms. Excessive excision of the cleft edges may give limited mucosa for closure and cause reduced interarytenoid space. In either technique of suturing, one should avoid *too tight* or *too loose* a closure that will cause a wound breakdown. Applying the correct pressure for tying the knot is surgeon dependent.
4. Wound healing after cleft repair (Fig. 4).

**Fig. 4:**
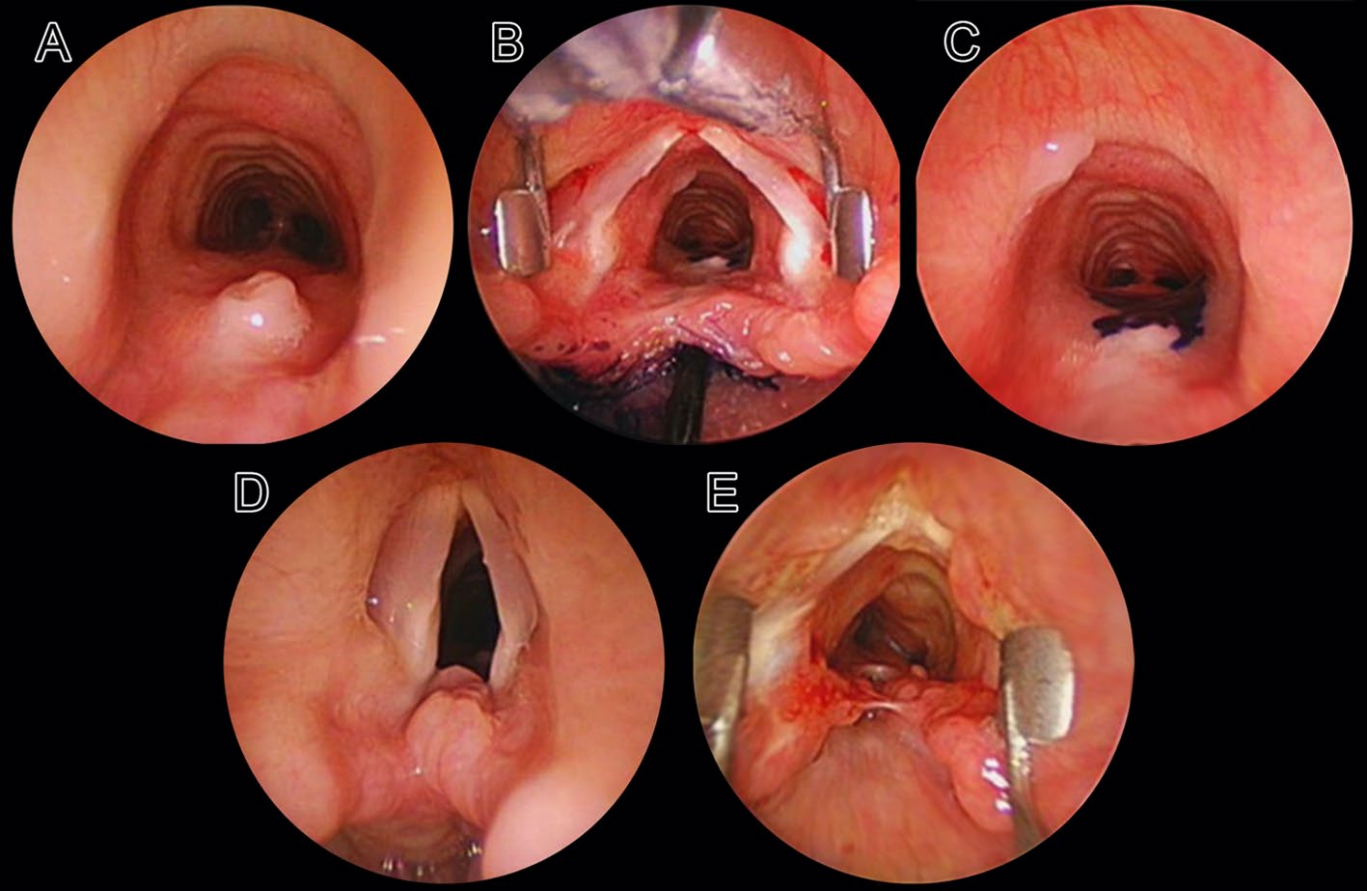
Non-optimal wound healing after laryngo-tracheo-esophageal Cleft (LTEC) repair. **A **Tracheoesophageal fistula (TEF). **B**, **C** Methylene Blue test showing passage of the dye through the TEF. **D** Proximal residual cleft. **E** Near-total breakdown
  Inadequate healing after a cleft repair can cause a residual cleft, tracheoesophageal fistula (TEF) or a complete breakdown. Caudally, the cleft mucosa must be removed up to the apex of the cleft and cranially, up to just below the cuneiform cartilages.
  Mucosa is a *non-stick surface* and therefore, inadequate mucosal denudation at the cleft apex will result in a TEF formation. In their 180 patients, Kou et al. [16] found that clefts having mucosal denudation done with CO2 laser had early breakdowns as compared to those repaired with cold steel.
  In our experience, one of the important aspects to avoid a cleft breakdown is to maintain an optimal interarytenoid space (IAS) and avoid its too tight closure that will restrict normal abduction movements of the arytenoids. Normal lateral movement of the arytenoids put strain on the suture line and may result in cleft recurrence. While creating the 2 distinct mucosal layers for a layered closure, it is critical to incise the cleft more posteriorly to make enough mucosa available for the repair and maintain an adequate IAS.
5. Additional technical points.
6. If a tracheostomy has to be done for a low-grade LTEC, the tracheal incision must be placed leaving at least 2 rings from the distal-most point of the cleft. This would avoid mechanical trauma by the cannula and stoma-related infection of the repair. Precise information regarding the length of the cannula necessary in a given case must be obtained by a rigid endoscopy prior to the placement of the tracheostomy, and reconfirmed after its placement by passing a flexible bronchoscope directly into the cannula. This will avoid damage to the carina by the cannula tip or its selective passing into one of the main stem bronchi. In longer clefts, the tracheostomy must be placed 7–10 days after the repair to avoid cannula-related decubitus effects on the suture line.
7. The ideal surgical approach for long clefts is still unclear. Laryngo-tracheo-fissure may cause laryngeal instability, induce cartilage damage and thereby malacia. Lateral pharyngotomy puts the recurrent laryngeal nerve at risk. For Type IV LTECs, Propst et al. [17] prefer waiting until the child attains 5 kg, then under extracorporeal membrane oxygenation support perform a cricotracheal separation to visualize the entire extent of the cleft for repair and then re-attach the cricoid to the trachea.

### Limitations

The present study has several limitations, the first being the limited sample size. However, the largest databases published are of similar size and is due to the rarity of the condition studied. Second, the length and quality of the follow-up are suboptimal and this is because most patients were referred to us from abroad. As a result, it was difficult to keep a standardized follow-up protocol and observations made by different doctors in different countries render the comparison of observations somewhat biased. This problem could be solved by developing a standardized questionnaire for the follow-up that is submitted at a fixed timeline to minimize the inter-observer bias. Third, its retrospective and observational study design.

## Conclusion

Patients with cleft extension up to couple of tracheal rings, without comorbidities, those who can withstand general anesthesia with spontaneous breathing and can be well exposed by suspension laryngoscopy can be successfully managed endoscopically. Longer clefts with long tracheal extension require an open approach. A comprehensive collaboration between various members of an airway team is critical for the overall treatment success.

## Data Availability

Not applicable.

## References

[1] Richter CF (1792) Dissertatio medica de infanticidae in artis obstetriciae exercitio non semper evitabili [doctor of medicine thesis]. Ex Officina Sommeria, Leipzig

[2] Leboulanger N, Garabédian E-N (2011). Laryngo-tracheo-oesophageal clefts. Orphanet J Rare Dis.

[3] Blumberg JB, Stevenson JR, Lemire KJ, Boxden EA (1965). Laryngotracheoesophageal cleft: the embryologic complications. Rev Literat Surg.

[4] Benjamin B, Inglis A (1989). Minor congenital laryngeal Clefts: diagnosis and classification. Ann Otol Rhinol Laryngol.

[5] Sandu K, Monnier P (2006). Endoscopic laryngotracheal cleft repair without tracheotomy or intubation. Laryngoscope.

[6] Rahbar R, Rouillon I, Roger G, Lin A, Nuss RC, Denoyelle F, McGill TJ, Healy GB, Garabedian EN (2006). The presentation and management of laryngeal cleft: a 10-year experience. Arch Otolaryngol Head Neck Surg.

[7] Leishman C, Monnier P, Jaquet Y (2014). Endoscopic repair of laryngotracheoesophageal clefts: experience in 17 cases. Int J Pediatr Otorhinolaryngol.

[8] Dodrill P (2015) Assessment of feeding and swallowing difficulties in infants and children. In: Groher M, Crary M (eds) Dysphagia: clinical management in adults and children (2e), 2015, Mosby

[9] Strychowsky JE, Dodrill P, Mortiz E, Perez J, Rabhar R (2016). Swallowing dysfunction among patients with laryngeal cleft: more than just aspiration?. Int J Pediatr Otorhinolaryngol.

[10] Kubba H, Gibson D, Bailey M, Hartley B (2005). Techniques and outcomes of laryngeal cleft repair: an update to the Great Osmond Street Hospital series. Ann Otol Rhinol Laryngol.

[11] Garabedian E-N, Pezzettigotta S, Leboulanger N, Harris R, Nevoux J, Denoyelle F, Roger G (2010). Endoscopic surgical treatment of laryngotracheal clefts. Arch Otolaryngol Head Neck Surg.

[12] Seidl E, Kramer J, Hoffmann F, Schön C, Griese M, Kappler M, Lisec K, Hubertus J, von Schweinitz D, Di Dio D, Sittel C, Reiter K (2021). Comorbidity and long-term clinical outcome of laryngotracheal clefts types III and IV: systematic analysis of new cases. Pediatr Pulmonol.

[13] Balakrishnan K, Cheng E, de Alarcon A, Sidell DR, Hart CK, Rutter MJ (2015). Outcomes and resource utilization of endoscopic mass-closure technique for laryngeal clefts. Otolaryngol Head Neck Surg.

[14] Benninger MS (2000). Microdissection or Microspot CO2 laser for limited vocal fold benign lesions: a prospective randomized trial. Laryngoscope.

[15] Remacle M, Lawson G, Nollevaux M-C, Delos M (2008). Current state of scanning micromanipulator applications with the carbon dioxide laser. Ann Otol Rhinol Laryngol.

[16] Kou Y-F, Kavoosi T, Redmann A, Manning A, Tabangin M, Myer CM, Hart CK, Rutter MJ, de Alarcon A (2021). Endoscopic repair of type 1 laryngeal clefts and deep interarytenoid notches: cold steel versus laser. Laryngoscope.

[17] Propst EJ, Ida JB, Rutter MJ (2013). Repair of long type IV posterior laryngeal cleft through a cervical approach using cricotracheal separation. Laryngoscope.

